# Relapse of Monomorphic Epitheliotropic Intestinal T‐Cell Lymphoma (MEITL) in a Pericardial Fluid

**DOI:** 10.1111/ijlh.14398

**Published:** 2024-11-17

**Authors:** Elsa Bera, Liana Veresezan, Maïssa Souissi, Fanny Drieux, Pierre Lebreton, Victor Bobée

**Affiliations:** ^1^ Department of Biological Hematology Rouen University Hospital Rouen France; ^2^ Department of Pathology Centre Henri Becquerel Rouen France; ^3^ Department of Hematology Centre Henri Becquerel Rouen France

**Keywords:** hematopathology, immunophenotype, lymphoma, MEITL

A 51‐year‐old man presented with bowel perforation and fecal peritonitis, necessitating bowel resection, ileostomy, and jejunostomy. Macroscopic examination revealed six flat, white tumors scattered along the jejunum and ileum, ranging in size from 5 to 9 cm, with a perforation present in the ileum. The histopathological examination of the intestinal resection showed a diffuse monomorphic lymphocytic infiltrate of atypical small‐ to medium‐sized lymphocytes (Figure 1A,B) with a T‐cell phenotype CD3^+^CD5^−^CD4^−^CD8^+^CD30^−^CD56^+^ weak TIA1^+^ Perforin–Granzyme B focal (Figure 1C). In situ hybridization using EBER probes was negative. The detection of a monoclonal rearrangement of the locus *TCRγ* and mutations of *SETD2* and *STAT5B* confirmed the diagnosis of Monomorphic Epitheliotropic Intestinal T‐cell Lymphoma (MEITL). The patient underwent chemotherapy (Brentuximab Vedotin, Cyclophosphamide, Doxorubicin, and Prednisone), achieving complete metabolic response, followed by an autologous stem cell transplant using the BEAM protocol (Bendamustine, Etoposide, Cytarabine, and Melphalan).

**FIGURE 1:**
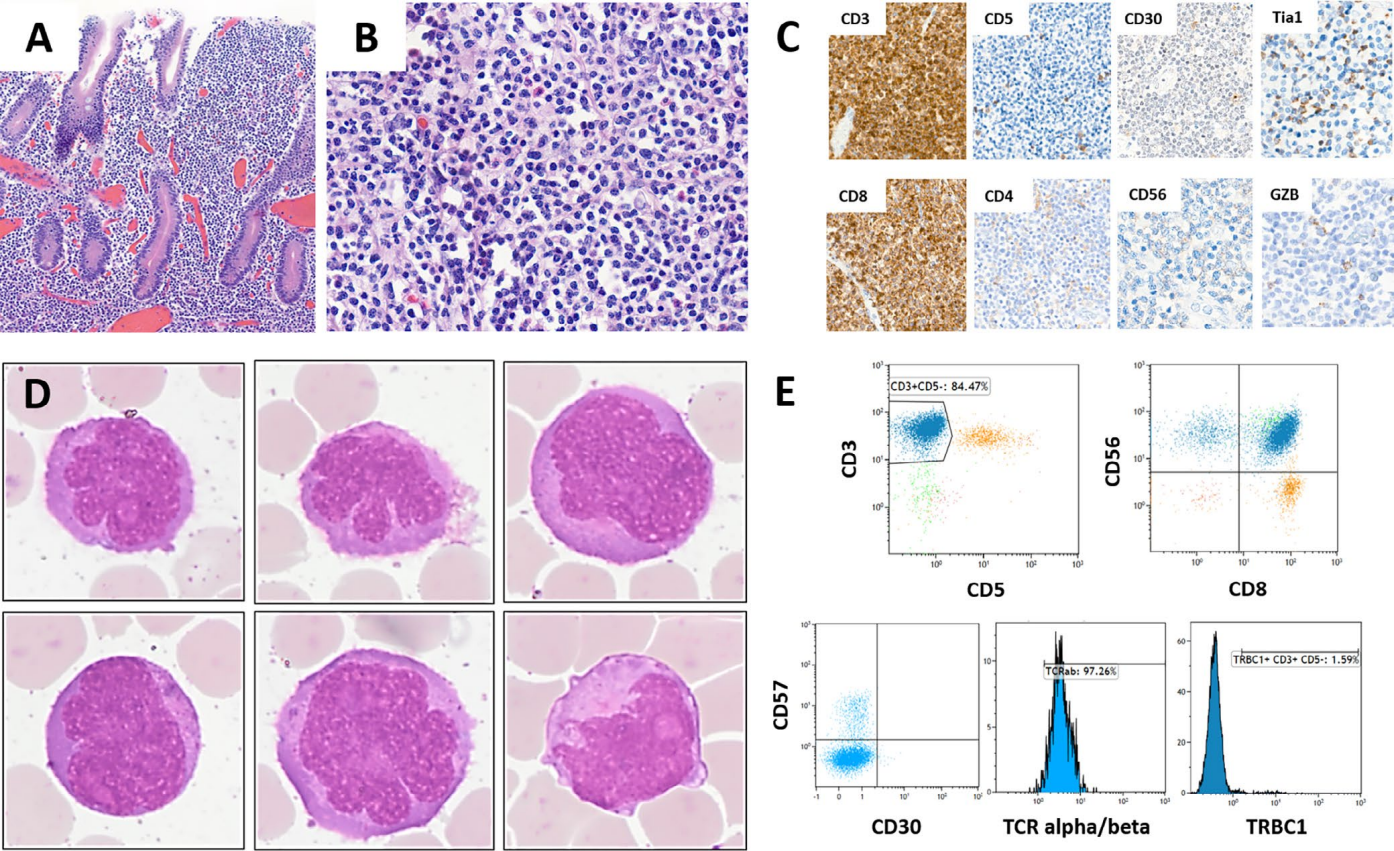
(A) Low magnification of one of the ileal tumors, with HE staining, revealed a transmural, diffuse, and monomorphic infiltrate. (B) The atypical lymphocytes were small to medium‐sized, with irregular nuclei and clear cytoplasm. (C) Immunohistochemistry panels showing a T‐cell phenotype CD3^+^CD5^−^CD4^−^CD8^+^CD30^−^CD56^+^ weak TIA1^+^ Granzyme B focal. (D) Pericardial fluid showing numerous large abnormal lymphocytes with large irregular nuclei and nucleoli, original magnification 100×, May–Grünwald Giemsa. (E) Flow cytometry analysis of pericardial fluid showing a CD3^+^CD5^−^CD8^+^CD56+ population of lymphocytes (blue) suggesting MEITL diagnosis. These cells have a TCRαβ and a restricted negative expression of TRBC1.

Seven months after transplantation, the patient presented with painful masses in the thoraco‐lumbar paravertebral region and left forearm, confirmed by PET‐Scan, and circumferential pericardial effusion. The cytologic analysis of the pericardial fluid revealed abnormal large cells with moderately condensed chromatin, irregular nuclei, nucleoli, and cytoplasmic granules (Figure 1D, May–Grünwald Giemsa, original magnification 1000×). Flow cytometry identified a contingent of abnormal CD3^+^CD5^−^CD8^+^CD56^+^ lymphocytes with TCRαβ phenotype and a restricted expression of TRBC1‐negative T cells (Figure 1E). A muscular biopsy of the left forearm confirmed the relapse of MEITL with the same immunophenotype. The tumor infiltrated the rectal, paraspinal, digestive, pulmonary, pericardic, and left forearm regions. Central nervous system (CNS) involvement was discussed without histological confirmation. The patient received a second‐line chemotherapy using bendamustine but unfortunately died 2 months later due to tumoral pneumopathy, 15 months after the initial diagnosis.

MEITL is a rare cytotoxic T‐cell lymphoma primarily affecting the gastrointestinal tract, following an aggressive course [1] with frequent CNS relapse [2]. Here, we report a case with atypical relapse sites, highlighting the critical role of histopathological, cytological examination, and flow cytometry, particularly in pericardial fluid, to achieve diagnosis and effective management.

## Author Contributions

E.B. performed research, analyzed data, and wrote the paper. M.S., F.D., L.V., and P.L. analyzed data. V.B. coordinated the study, wrote the paper, and supervised the analysis.

## Consent

No written consent has been obtained from the deceased patient, as there is no patient‐identifiable data included in this case report.

## Conflicts of Interest

The authors declare no conflicts of interest.

## Data Availability

All data are available from the corresponding author upon request.
